# Lead concentration in follicular fluid of infertile patients with and without endometriosis: a propensity score matching exploratory study

**DOI:** 10.3389/fendo.2026.1814365

**Published:** 2026-05-22

**Authors:** Irene Iavarone, Daniela Mele, Francesca Caprio, Buenaventura Coroleu, Pasquale De Franciscis, Luigi Cobellis, Carlo Ronsini

**Affiliations:** 1 Department of Woman, Child and General and Specialized Surgery, University of Campania Luigi Vanvitelli, Naples, Italy; 2 Dexeus Fertility, Department of Obstetrics Gynecology and Reproductive Medicine, Hospital Universitari Dexeus, Barcelona, Spain; 3 Unit of Gynecologic Oncology, National Cancer Institute, IRCCS, Fondazione “G. Pascale”, Naples, Italy

**Keywords:** endocrine disruptors, endometriosis, follicular fluid, infertility, lead exposure

## Abstract

**Objective:**

This preliminary study sought to provide initial insights into lead concentrations within the follicular fluid of infertile patients with and without endometriosis, tentatively observing any potential links between lead presence and the condition. Furthermore, we attempted to discern whether these levels might show a possible correlation with *In Vitro* Fertilization (IVF) outcomes.

**Methods:**

We conducted an exploratory retrospective observational case-control study at the Fertility Unit of the University of Campania Luigi Vanvitelli, utilizing propensity score matching to compare follicular fluid samples from infertile patients with and without endometriosis undergoing IVF from January 2022 to July 2024. The study’s initial aim was to tentatively assess variations in lead concentration in the follicular fluid of women undergoing IVF with or without endometriosis. Additionally, a small-scale sub-analysis was performed to begin investigating how these follicular lead levels might relate to serum Beta-Human Chorionic Gonadotropin (hCG) positivity after embryo transfer in the endometriosis group.

**Results:**

In this preliminary evaluation, follicular fluid samples from 46 patients were analyzed, comprising 13 with endometriosis and 33 matched controls without. The median follicular lead concentration was notably higher in patients with endometriosis (2.07 µg/dL) compared to controls (0.63 µg/dL; p<0.001). Logistic regression analysis suggested a possible association between elevated lead levels and increased odds of endometriosis (OR: 14.6, 95% CI: 2.50–142.59, p = 0.007). In our further exploratory sub-analysis of endometriosis patients, 30.8% had positive serum Beta-hCG after embryo transfer but the variation in follicular lead concentration between positive serum Beta-hCG (1.00 µg/dL) and negative (2.54 µg/dL) was not statistically significant (p=0.12).

**Conclusions:**

While our exploratory observations within this limited cohort suggest higher lead levels in the follicular fluid of patients with endometriosis compared to those without, these initial findings should be considered hypothesis-generating. Given the modest sample size, independent validation through larger, robustly designed prospective studies is essential.

## 1 Introduction

Endometriosis has garnered increasing attention in recent years due to its potential impact on women’s reproductive health (1). One significant area of concern is the role of environmental contaminants, particularly heavy metals, in developing and exacerbating endometriosis. Lead, classified as an endocrine disruptor, has been implicated in various reproductive disorders, raising questions about its contribution to the pathophysiology of endometriosis (2). The endocrine-disrupting properties of lead can interfere with hormonal regulation, potentially influencing the growth of endometrial tissue and its associated symptoms (3).

Moreover, the link between endometriosis and infertility is well-established, with studies indicating that women suffering from this condition face significantly higher rates of infertility compared to those without it (4). Conversely, lead can itself negatively influence various reproductive outcomes, including ovulatory function and sperm quality in men (5). It is conceivable that a complex interdependence exists between these three conditions. Consequently, further exploratory efforts focusing on lead, endometriosis, and infertility might offer preliminary glimpses into the shared pathways that potentially link these factors.

Evidence regarding the impact of environmental contaminant exposure on infertility remains limited and extends beyond endometriosis to encompass other reproductive disorders, such as polycystic ovary syndrome (PCOS) (6). The current literature is further complicated by the fact that reduced ovarian reserve has also been associated with toxicant exposure, creating a degree of ambiguity in isolating specific effects. Regarding endometriosis, data remains particularly scarce, even though its pathogenesis is widely hypothesized to be linked to the action of endocrine disruptors (7, 8).

This study seeks to explore variations in lead levels within the follicular fluid of infertile patients, with and without endometriosis, aiming to observe any potential links between lead presence and the condition. Furthermore, we attempted to discern how these concentrations might be associated with Assisted Reproduction Techniques (ART) outcomes, specifically *In Vitro* Fertilization (IVF). By examining these initial connections, we aspire to offer an insight to the understanding of the possible relationship between environmental toxins, endometriosis, and infertility.

## 2 Materials and methods

### 2.1 Study design

We conducted an exploratory, retrospective observational case-control study by propensity score matching of patients with infertility and co-presence or absence of endometriosis who underwent IVF at the Infertility Centre of the University of Campania Luigi Vanvitelli. The study was conducted according to the STROBE statement for observational studies (9). The study was conducted in a university clinic where all patients treated must sign a dedicated consent for anonymous data processing, and one specific to the study. According to the regulations in force in the state where the study was conducted, IRB from the local Ethical Committee was not required due to the retrospective nature of the analysis.

Initial outcome of the study was to exploratively observe whether differences exist in follicular fluid lead concentrations among women undergoing IVF, comparing those with and without endometriosis. Additionally, we performed a preliminary sub-analysis focused specifically on patients with endometriosis to identify potential observational associations between follicular lead levels and serum Beta- Human Chorionic Gonadotropin (hCG) positivity following embryo transfer.

### 2.2 Setting and IVF procedure

In this exploratory analysis, all samples from patients undergoing IVF at the Fertility Unit of the University of Campania Luigi Vanvitelli between January 2022 and July 2024 underwent follicular lead assay. 13 of these patients had a histological diagnosis of endometriosis and were included as cases. The patients underwent ovarian stimulation following a flexible Gonadotropin-Releasing Hormone (GnRH) antagonist protocol. Both cases and controls received identical stimulation with 150 to 300 IU of recombinant Follicle-Stimulating Hormone (FSH) starting on day 2 or 3 of their menstrual cycle. Daily GnRH antagonist injections were administered to prevent premature ovulation from the day the leading follicle reached a 14mm diameter until the hCG injection. Ovulation was monitored through transvaginal ultrasound and hormonal assessments every two days. When at least two follicles reached a diameter of 18mm, a single intramuscular dose of 10,000 IU of hCG (Gonasi HP 5000; IBSA, Rome, Italy) was administered. Follicular aspiration was performed 34 to 36 hours after the hCG injection and lead dosage was directly conducted. All patients underwent Intracytoplasmic Sperm Injection (ICSI) and received an embryo-transfer by the physician’s decision between day 4 and day 6.

### 2.3 Participants

Inclusion criteria were: Complete information regarding follicular lead assay at the time of Pick-Up; Complete information regarding age and Body Mass Index (BMI) at the time of IVF; Complete information regarding the feasibility of the embryo-transfer technique; Histological diagnosis of endometriosis for patients included as cases; Absence of clinical suspicion of endometriosis for patients included as controls. Exclusion criteria were chronic pelvic pain of unknown nature in controls, dysmenorrhea ≥ 6/10 according to Visual Analogue Scale (VAS) in controls, and occupational activities with lead exposure (metallurgy sector, construction sector).

### 2.4 Variables

The variables examined were age, expressed as a continuous variable in years; BMI as a continuous variable in Kg/m^2^; the cause of infertility expressed as a clinical diagnosis and considered as an ordinal variable; the diagnosis of endometriosis, understood as a histological diagnosis of certainty following surgery, and considered as a dichotomous variable; Follicular lead concentration, expressed in µg/dL as a continuous variable and considered primary initial outcome; The pregnancy variable was considered as a dichotomous variable based on Beta-hCG positivity 14 days after the embryo transfer and used to stratify the sample in the further preliminary sub-analysis. Endometriosis was stratified based on stage according to the American Society of Reproductive Medicine (ASRM) (10).

### 2.5 Propensity score matching and risk of bias

To minimize the presence of confounders, we conducted a preliminary matching of the 13 patients with infertility and endometriosis at a 1:3 ratio with those not affected by the condition fulfilling inclusion criteria. The variables on which the exploratory propensity score matching was performed were age, BMI and cause of infertility (tubal, ovarian or male factor), to help address factors that might influence serum lead distribution, linked to different BMI and consequently a hypothetical different follicular distribution, possible age-related years of exposure and success rate of reproductive technique. 33 patients met the inclusion criteria and were included based on these characteristics. They thus formed the control group. All patients were managed within a standardized clinical protocol, including controlled ovarian stimulation, uniform laboratory procedures, and pre-treatment clinical optimization. These measures tentatively contributed to mitigating the unavoidable variability of treatment-related and lifestyle factors. No missing data were present.

### 2.6 Laboratory dosage

At the time of Pick-Up, a sample of 10 mL of follicular fluid was retrieved from each ovary. Samples were stored at the temperature of -20 C until analysis. A K2EDTA Vacutainer^®^ 5 mL tube was used for the lead determination. All biological samples were transported to the laboratory in thermal bags within 4 hours from the collection and analyzed within a day. Follicular concentrations of metals were determined by flame atomic double beam absorption spectrometry with a graphite furnace (Analyst 800, PerkinElmer, Italy). The follicular fluid samples were 1:20-diluted with Triton X-100 0,1% to identify the lead level.

### 2.7 Statistical analysis

The *null* hypothesis of our exploratory analysis was that there was no difference in the median values of Lead in the follicular fluid of infertile patients with and without endometriosis (H0: μ1=μ2). The distribution of the continuous variables for this parameter of the reference outcome was graphed in boxplots. Continuous variables were expressed as median and interquartile range and compared using the Kruskal-Wallis test due to the nonparametric distribution (11).

In the preliminary sub-analysis, the sample was stratified according to the pregnancy outcome.

The nominal variables were expressed as absolute frequency and percentages. The association between follicular lead concentration and binary outcomes (presence of endometriosis; biochemical pregnancy) was evaluated using univariable logistic regression models, with results reported as odds ratios (ORs) and 95% confidence intervals (CIs) (12). The significance of the model used was assessed using the maximum likelihood method (13). Given the limited sample size, particularly in the subgroup analyses, no formal sample size calculation was performed. Consequently, our evaluations are purely exploratory and observational, highlighting statistical associations within our cohort and not being interpreted as evidence of a causal relationship.

The statistical significance level was set at 0.05, and all statistical investigations were performed using R software and R Studio vers. 2023.12.1 + 402. Propensity score matching was performed using R’s ‘MatchIt’ package, with a 1:3 ratio, using the ‘nearest neighbor’ method and a caliper set to 0.1.

## 3 Results

In this exploratory analysis, 13 samples from patients suffering from infertility and endometriosis were retrospectively tested for follicular lead concentrations during Pick-Up. These were matched at a 1:3 ratio with samples from a control group of 33 infertile patients undergoing Pick-Up. The control group was constructed based on median age, BMI, and cause of infertility. According to the ASRM classification (7), the endometriosis group consisted of 8 (61.5%) patients with stage I or II endometriosis and 5 (38.5%) patients with stage ≥ 3. The characteristics of the test population are summarized in **Table 1**.

**Table 1:** Patients’ characteristics after propensity score matching.

Characteristic	Non-endometriosis, N=33^1^	Endometriosis, N=13^1^
Age	35.8, (7.0)	35.3, (6.0)
BMI	23.4, (7.0)	23.5, (5.0)
Other infertility factors		
Male	18, (55%)	7, (54%)
Ovarian	8, (24%)	4, (31%)
Tubal	7, (21%)	2, (15%)
Endometriosis		
Stage I-II^3^	-	8, (61.5%)
Stage III-IV^3^	-	5, (38.5%)

BMI, Body Mass Index.

^1^ Median, (IQR); n, (%).

^2^ Wilcoxon rank sum test; Fisher’s exact test.

^3^ ASRM staging system.

### 3.1 Outcomes

**Figure 1:**
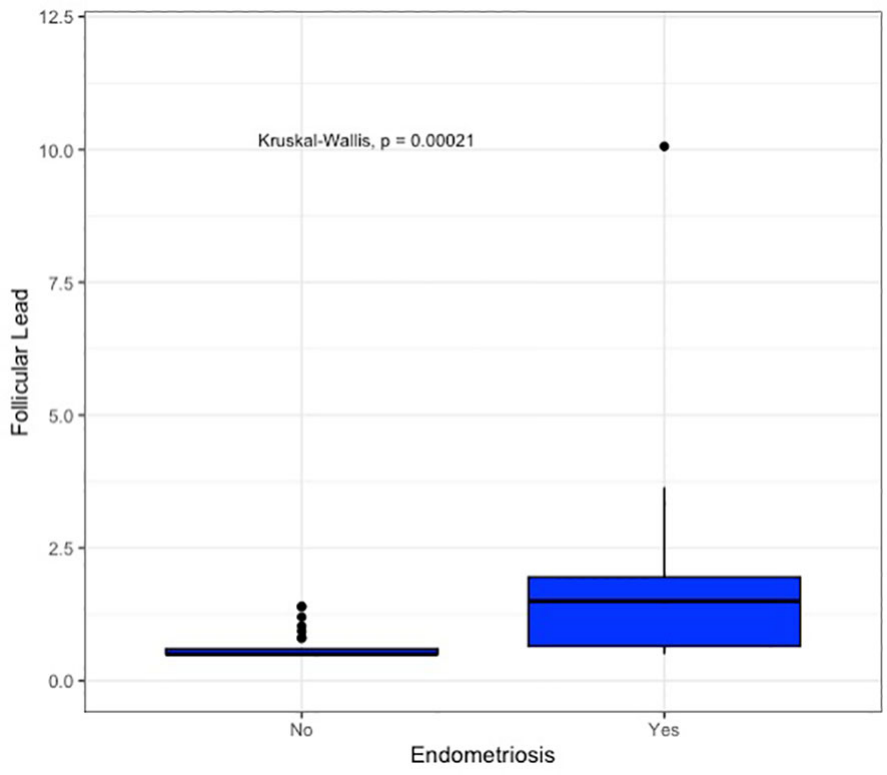
Box plot representing follicular lead concentration in controls and cases.

The primary outcome of this exploratory analysis was to observe potential variations in lead concentration within the follicular fluid of infertile patients, comparing those with and without endometriosis. The follicular lead concentration appeared significantly higher in patients with endometriosis (Follicular Lead 2.07 vs. 0.63; p<0.001). This result is summarized in **Table 2** and graphed by box plot in **Figure 1**.

**Table 2:** Outcomes.

Characteristic	Non-endometriosis, N=33^1^	Endometriosis, N=13^1^	p-value^2^
Follicular Lead^3^	0.63, (0.10)	2.07, (1.30)	**<0.001**

^1^ Median, (IQR).

^2^ Wilcoxon rank sum test.

^3^ µg/dL.

Bold values indicate statistical significance. The statistical significance level was set at 0.05.

To exploratively investigate the potential observational association between follicular lead concentration and the presence of endometriosis, we performed a univariable logistic regression model, considering endometriosis as a binary dependent variable. Results were reported as ORs with 95% CIs.

It was observed that higher follicular lead concentration was found to have a statistically significant association with increased odds of endometriosis (OR: 14.6, 95% CI: 2.50–142.59, p = 0.007), as represented in **Table 2.1**.

**Table 2.1:** Logit regression analysis.

Endometriosis						
Variable	Estimate	Standard Error	z value	p-value	OR^1^	OR 95% CI^2^
Follicular Lead^3^	2.681	1.002	2.676	0.007	14.6	2.502 - 142.594

^1^ OD, Odds Ratio.

^2^ CI, Confidence Interval.

^3^ µg/dL.

**Table 3:** Biochemical pregnancy in endometriotic patients.

Characteristic	Biochemical pregnancy		p-value^2^
	No, N=9^1^	Yes, N=4^1^	
Follicular Lead^3^	2.54, (1.13)	1.00, (0.95)	0.12

^1^ Median, (IQR).

^2^ Wilcoxon rank sum test.

^3^ µg/dL.

### 3.2 Sub-analysis of endometriotic patients

**Figure 2:**
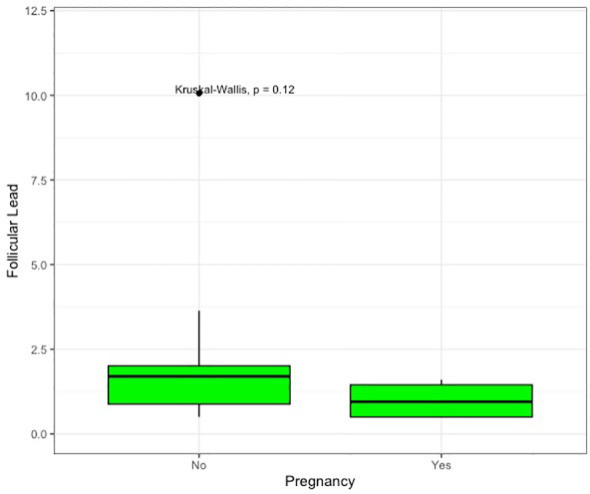
Box plot of follicular lead concentration in endometriotic patients based on the positivity of serum beta-hCG after embryo transfer.

We conducted an exploratory sub-analysis in endometriosis patients only, stratifying the sample based on biochemical pregnancy. Of the 13 patients, 4 (30.8%) showed positive Beta-hCG after embryo transfer. While follicular lead levels appeared higher in cases with negative Beta-hCG, this difference was not statistically significant (2.54 vs. 1.00; p=0.12). Those preliminary observations are represented in **Table 3** and graphed in **Figure 2**.

In the preliminary sub-analysis restricted to endometriosis patients, a univariable logistic regression model was performed to observe potential links between follicular lead concentration and biochemical pregnancy. No statistically significant association was observed (OR: 0.46, 95% CI: 0.015–7.44, p = 0.58). The notably wide CI highlights the unavoidable limitations of our small sample size and few events, as shown in **Table 3.1**.

**Table 3.1:** Logit regression analysis of follicular lead concentration in endometriotic patients based on the positivity of serum beta-hCG after embryo transfer.

Endometriosis						
Variable	Estimate	Standard Error	z value	p-value	OR^1^	OR 95% CI^2^
Follicular Lead^3^	-0.783	1.420	-0.551	0.581	0.457	0.015 - 7.441

^1^ OD, Odds Ratio.

^2^ CI, Confidence Interval.

^3^ µg/dL.

## 4 Discussion

Our exploratory research observed that the follicular fluid of infertile endometriosis patients appears to have higher average lead concentrations. The logistic regression model suggested an observational association between these variables, though it is important to note that this does not constitute confirmatory evidence. As an exploratory and observational study, our findings do not imply a causal inference. Indeed, an important limitation of the present study is the relatively small sample size, particularly in the endometriosis group and, more markedly, in the subgroup of patients achieving biochemical pregnancy. This aspect may limit the statistical power of the regression analyses and contribute to the wide confidence intervals observed, especially in the IVF outcome analysis.

Our sub-analysis regarding biochemical pregnancy did not identify a statistically significant relationship with IVF outcome. Although a difference in median values was observed, the non-significant effect and wide confidence intervals observed highlight substantial imprecision, and these results are intended solely for hypothesis-generating purposes rather than providing confirmatory evidence. No conclusions regarding the impact of follicular lead concentration on pregnancy outcome can be drawn from the present data and should be interpreted with caution.

Even though the standardized preliminary assessment implemented at our center – smoking cessation and nutritional counseling – attempted to address procedural consistency, these measures cannot mitigate unavoidable residual confounding inherent to this research. Factors such as uncontrolled environmental exposure, lifestyle variables, and disease heterogeneity represent residual confounders.

Moreover, while propensity score matching addressed age, BMI, and infertility cause, other potential confounders – as lifestyle (e.g., smoking), environmental variability, ovarian reserve parameters, stimulation characteristics, and embryo quality – were not included in the matching process. Due to the small sample size, no further statistical adjustments were made to prevent model instability. Consequently, this exploratory observational study identifies associations that should not be interpreted as causal, also considering the additional limitation of its retrospective nature.

Our exploratory findings align with existing literature regarding lead exposure and endometriosis. As an endocrine disruptor, lead hypothetically could parallel the growth and regulation of endometrial tissue, potentially contributing to the development or progression of the disease. Furthermore, lead contamination may negatively affect fertility by impairing ovarian function and compromising oocyte quality. However, we must emphasize that this exploratory study identifies strictly observational associations rather than any definitive causal link. Consequently, women with endometriosis may represent a vulnerable population due to these potential, though not yet causally proven, combined effects. Recently Kim et al. (14) showed that individuals exposed to lead and cadmium have a higher incidence of endometriosis. Schiattarella et al. showed that in patients with severe endometriosis, plasma and urinary lead concentrations differed from the general population (15).

Previous research has consistently demonstrated a correlation between endometriosis and exposure to heavy metals, also with particular focus on lead. In 2013, Singh et al. demonstrated that patients with endometriosis exhibited a significant imbalance in follicular biomarkers, characterized by an elevation in oxidative stress markers and heavy metals – iron, lead, and cadmium –, along with a marked depletion of antioxidants, including SOD, catalase, and essential vitamins – A, C, E (16). In addition, a case presentation with no history of exposure documented a peritoneal endometriosis with increased lead, nickel, and bismuth concentrations in the peritoneal fluid, suggesting that the levels of these disruptors may parallel the pathogenesis of the disease (17). Finally, a Chinese study in 2023 demonstrated the significant correlation of the serum levels of arsenic, cadmium, lead, and mercury with the increased risk of endometriosis in a multivariate analysis (18).

Specifically, the evidence that high concentrations of lead can be found in the follicular fluid of women with endometriosis forces us to make clinical considerations in the management of these patients. Endometriosis is known to have multiple factors influencing its onset and progression, many of which are environmental (19). Indeed, correction of diet and lifestyle may be a necessary step in optimizing the management of these patients. Recently, it has been highlighted that the type of gut microbiota is also different in patients with endometriosis (20). In this scenario, consuming foods rich in endocrine disruptors could exacerbate endometriosis disease (21), which is also based on the correlation highlighted by this study. In addition, the increased presence of lead in follicular fluid, should the correlation with IVF outcomes be confirmed, the mechanisms by which this occurs would need to be investigated, both from a response to therapy perspective and from a teratogenic risk to the embryo perspective, even in light of the rising of Preimplantation Genetic Testing for Aneuploidies (PGT-A) (22).

We hypothesize a speculative bidirectional link where endocrine disruption might exacerbate the disease, while inflammatory states could potentially facilitate lead accumulation. These are potential directions for deeper longitudinal investigation. A starting point for translational research could be investigating how the chronic inflammatory state inherent to endometriosis might facilitate lead accumulation in affected tissues through macrophage action. On the other hand, among future perspectives in clinical research, it would be helpful to enroll a control sample of healthy, fertile women, to contextualize lead concentrations more broadly and enhance the generalizability of findings. This poses serious ethical problems in the sampling of follicular fluid from healthy women who do not have to undergo oocyte pick-up for infertility reasons. In addition, further studies would experimentally assess whether reducing lead exposure impact endometriosis severity or IVF outcomes, providing stronger evidence for clinical recommendations.

## 5 Conclusions

In this small exploratory cohort, an observational association was identified between follicular lead concentrations and endometriosis. These preliminary findings require independent validation in larger, well-designed studies to determine their significance.

## Data Availability

The raw data supporting the conclusions of this article will be made available by the authors, without undue reservation.
